# Occupational class and male cancer incidence: Nationwide, multicenter, hospital‐based case–control study in Japan

**DOI:** 10.1002/cam4.1945

**Published:** 2019-01-04

**Authors:** Masayoshi Zaitsu, Rena Kaneko, Takumi Takeuchi, Yuzuru Sato, Yasuki Kobayashi, Ichiro Kawachi

**Affiliations:** ^1^ Department of Social and Behavioral Sciences Harvard T.H. Chan School of Public Health Boston Massachusetts; ^2^ Department of Public Health, Graduate School of Medicine The University of Tokyo Tokyo Japan; ^3^ Department of Gastroenterology Kanto Rosai Hospital Kawasaki Kanagawa Japan; ^4^ Department of Urology Kanto Rosai Hospital Kawasaki Kanagawa Japan

**Keywords:** cancer incidence, Japan, occupation, risk, socioeconomic status

## Abstract

Little is known about socioeconomic inequalities in male cancer incidence in nonwestern settings. Using the nationwide clinical and occupational inpatient data (1984‐2016) in Japan, we performed a multicentered, matched case–control study with 214 123 male cancer cases and 1 026 247 inpatient controls. Based on the standardized national classifications, we grouped patients’ longest‐held occupational class (blue‐collar, service, professional, manager), cross‐classified by industrial cluster (blue‐collar, service, white‐collar). Using blue‐collar workers in blue‐collar industries as the referent group, odds ratios (ORs) and 95% confidence intervals (CIs) were estimated by conditional logistic regression with multiple imputation, matched for age, admission date, and admitting hospital. Smoking and alcohol consumption were additionally adjusted. Across all industries, a reduced risk with higher occupational class (professionals and managers) was observed for stomach and lung cancer. Even after controlling for smoking and alcohol consumption, the reduced odds persisted: OR of managers in white‐collar industries was 0.80 (95% CI 0.72‐0.90) for stomach cancer, and OR of managers in white‐collar industries was 0.66 (95% CI 0.55‐0.79) for lung cancer. In white‐collar industries, higher occupational class men tended to have lower a reduced risk for most common types of cancer, with the exception of professionals who showed an excess risk for prostate cancer. We documented socioeconomic inequalities in male cancer incidence in Japan, which could not be explained by smoking and alcohol consumption.

## 1 INTRODUCTION

Cancer is a leading cause of death in developed countries, and in 2016, the total incidence of cancer was estimated to be 867 408 (male 501 527 and female 365 881) in Japan.^1^ Although overall cancer mortality has been declining in Japan, where stomach cancer appeared to play a large role for the decrease due to improved risk factors (eg smoking, salt intake, and *Helicobacter pylori* infection) and treatment strategies, overall cancer incidence has been continuously increasing.^2^

In Western countries, occupational class, a fundamental proxy for socioeconomic status (SES), is considered as a major determinant of cancer incidence.^3^ For example, stomach and lung cancers tend to show a reduced risk in higher‐SES individuals, such as professional and managerial workers.^3^ Major lifestyle risk behaviors, such as smoking and alcohol consumption, are thought to underlie the observed socioeconomic gradient in cancer risk.^3^ For example, smoking is less prevalent in higher occupational class, and this may account for a lower risk of stomach and lung cancer.^4^, ^5^

In Japan, as well as in other Asian countries, although previous studies investigated the association between occupational class and cancer *mortality* (but not incidence)^6^, ^7^ or the ecological association between cancer incidence and *regional‐level* SES (but not individual level),^8^, ^9^ few studies evaluated the association of occupational class and risk of cancer incidence using individual‐level data. Also, the background cancer risks associated with occupational class differ between western and nonwestern contexts. For example, compared with Western countries, the distribution of *H. pylori* infection (stomach cancer risk) is higher in Japan.^2^, ^10^ For socioeconomic patterns for other potential cancer risks related to occupation, work‐related psychological stress partly differ between these two contexts.^11^ In contrast to Western countries, where occupational stress is typically higher among low‐occupational classes compared with high‐occupational ones, the opposite pattern has been seen in Japan (eg high suicide rate in managerial position).^6^, ^11^ Recently, with regard to major cancer incidence among women in Japan, we found a reduced risk of stomach and lung cancer and an excess risk of breast cancer in higher occupational class using individual‐level data.^10^ However, the association among men remains unclear in Japan. As applying female results to men is inappropriate due to etiology of cancer^12^ and distribution of occupational class,^6^ it is necessary to determine socioeconomic inequalities in male cancer incidence separately from those with females.

Using a nationwide, multicenter inpatient dataset including individual‐level clinical data and occupational information, we examined whether the risk of male cancer incidence is associated with occupational class in Japan. We also determined whether the observed association persists even after controlling for smoking and alcohol consumption.

## 2 MATERIALS AND METHODS

### 2.1 Study setting

We conducted a multicenter, hospital‐based matched case–control study using male inpatient data from the Inpatient Clinico‐Occupational Database of Rosai Hospital Group (ICOD‐R), run by the Japan Organization of Occupational Health and Safety (JOHAS). Details of ICOD‐R have been described elsewhere.^10^, ^11^, ^13^, ^14^, ^15^ Briefly, the Rosai Hospital group consists of 33 general hospitals in main urban areas and rural areas of Japan; it has collected medical chart information confirmed by physicians (including basic socio‐demographic characteristics, clinical history, and diagnosis of current and past diseases, pathological information, treatment, and outcome for every inpatient) since 1984. The clinical diagnosis and comorbid diseases, extracted from physicians’ medical charts confirmed at discharge, are coded according to the International Classification of Diseases, 9th Revision (ICD‐9) or 10th Revision (ICD‐10).^10^, ^11^, ^13^, ^14^, ^15^ Although the Rosai Hospitals were initially established by the Ministry of Labour of Japan in 1949 for the working population, the hospital group has since expanded coverage to the general population as well as the working population.^14^ The profiles of the patients, including occupational class, are nationally representative.^10^, ^11^

From questionnaires completed at the time of admission, ICOD‐R also includes the occupational history of every inpatient (current and three most recent jobs with duration) as well as smoking and alcohol habits (status, daily amount, and duration). Detailed occupational history is coded with the three‐digit codes of the standardized national classification, the Japan Standard Occupational Classification and Japan Standard Industrial Classification, corresponding, respectively, to the International Standard Industrial Classification and International Standard Occupational Classification; JOHAS updated the previous job codes to be consistent with changes in coding practice according to the revisions of the standardized national classification.^10^, ^11^, ^13^, ^14^, ^15^ Written informed consent was obtained before patients completed the questionnaires; trained registrars and nurses are in charge of registering the data. The database currently contains data from over 6 million inpatients.

We obtained a de‐identified dataset under the research agreement between the authors and JOHAS, and the research ethics committees of The University of Tokyo, Tokyo (Protocol Number 3890‐5) and Kanto Rosai Hospital, Kanagawa (Protocol Number 2014‐38) approved the study.

### 2.2 Cases and controls

The study subjects comprised 1 240 370 subjects (214 123 male cancer cases and their 1 026 247 male hospital controls) aged 20 years and older admitted to the hospital between 1984 and 2016. To select cases and controls from the same source population, we randomly sampled five controls for each cancer case, matched for age, admission date, and admitting hospital.^10^, ^14^ The matching process, however, generated less than five controls for some cases.

The cancer cases comprised those patients whose main diagnosis was initial cancer, confirmed by physicians on discharge with their medical chart information, pathological, or imaging information (computed tomography, magnetic resonance imaging, and endoscopy).^10^, ^11^, ^13^, ^14^, ^15^ We defined cancer incidence as the first‐time admission to the hospitals with a cancer diagnosis; the validation for the diagnosis corresponding to ICD‐9 or ICD‐10 in the database has been described elsewhere.^10^, ^11^, ^13^, ^14^, ^15^ The database is unique to the Rosai Hospital group and so differs from medical claims data, which may have less diagnostic accuracy.^16^ Following national statistics in Japan,^1^, ^17^, ^18^ we specified the top 10 common male cancer sites: stomach, lung, colorectum, prostate, liver, esophagus, pancreas, bladder, kidney (including pelvis and ureter), and malignant lymphoma (Table S1). Less common cancers were additionally specified. The prevalence of these cancers was mostly identical to that in national statistics, and the total of our male cancer cases amounted ~2% of the total incidence of male cancer in Japan (Table S1).^1^, ^17^, ^18^

Based on a methodology used in previous studies,^10^, ^11^ our controls comprised male patients diagnosed with eye and ear disease (ICD‐9, 360‐389 and ICD‐10, H00‐H95; 36.5%), genitourinary system disease (ICD‐9, 580‐629 and ICD‐10, N00‐N99; 42.9%), infectious and parasitic disease (ICD‐9, 1‐136 and ICD‐10, A00‐B99; 13.6%), or skin diseases (ICD‐9, 680‐709 and ICD‐10, L00‐L99; 7.0%), which were not linked to occupational class (Figure S1).

### 2.3 Occupational class and covariates

To classify occupational class, we chose the longest‐held job for each patient from his occupational history (current and three most recent jobs).^10^, ^11^ The longest‐held occupations were classified into four occupational classes (blue‐collar, service, professional, and manager), cross‐classified by three industrial clusters (blue‐collar industry, service industry, and white‐collar industry; Figure S2).^10^, ^11^ That is, the blue‐collar industry included agriculture, forestry and fisheries, mining and quarrying of stone, construction, manufacturing, electricity, gas, heat supply and water, and transport and postal services; the service industry included wholesale and retail trade, accommodations, eating and drinking services, living‐related, personal and amusement services, compound services, and services not elsewhere categorized; and the white‐collar industry included information and communications, finance and insurance, real estate, goods rental and leasing, education and learning support, medical, health care and welfare, and government except elsewhere classified.^10^, ^11^ The “other” group comprised patients who were not actively engaged in paid employment (unemployed, nonworker, miscellaneous worker, and student) were additionally specified.

Confounding factors included age, admission date, and admitting hospitals, and mediating factors included smoking (log [1 + pack‐year]) and alcohol consumption (log [1 + daily gram of ethanol intake]).^10^, ^11^, ^13^, ^14^ Drinking habits were assessed prior to symptom onset related to admission.

### 2.4 Statistical analysis

Overall one‐third of the study subjects had missing data, and excluding those with missing data may lead to biased inference.^11^ To deal with missing data, we performed multiple imputation for missing data among 1 240 370 study subjects using all data, including occupational class, smoking, and alcohol consumption.^10^, ^11^, ^19^ Five imputed datasets with Multiple Imputation by Chained Equations method were generated.^10^, ^11^, ^19^ The following missing data were multiply imputed: occupational class (350 751, 28.3%), smoking (385 511, 31.1%), alcohol consumption (478 059, 38.5%).^10^, ^11^

Next, using blue‐collar workers in blue‐collar industries as the referent group, odds ratios (ORs), and 95% confidence intervals (CIs) in each occupational class for specific cancer sites and overall cancer incidence were estimated by conditional logistic regression with multiple imputation, matched for age, admission date, and admitting hospital (model 1).^10^, ^11^, ^19^ To assess the contribution of major modifiable risk factors, smoking and alcohol consumption were additionally adjusted (model 2).

In sensitivity analyses, based on the distribution of our data and previous studies from ICOD‐R, we performed stratified analyses by age (20‐64 vs 65 and above) and admission date (1984‐2002 vs 2003‐2016), respectively.^13^, ^20^ In addition, without performing multiple imputation, we performed (a) conditional logistic regression and (b) multilevel logistic regression with random intercepts fitted for each hospital (level 1, individual; level 2, hospital), among patients with complete information (125 342 cases, 559 198 controls). Due to insufficient number of the cases, these analyses were limited to stomach, lung, prostate, and overall cancer. Additionally, using alternative control groups (all available hospital controls diagnosed with benign diseases), we performed conditional logistic regression with multiple imputation for lung cancer (22 086 cases, 110 321 controls) and prostate cancer (28 648 cases, 143 090 controls). Alpha was set at 0.05, and all *P*‐values were two‐sided. Data were analyzed using STATA/MP13.1 (Stata‐Corp LP, College Station, TX).

## 3 RESULTS

The mean age [mean (SD)] in the controls and cases was, respectively, 67 (11) years and 67 (11) years. Higher occupational class was clearly associated with reduced risks for stomach and lung cancer. In all three industries, higher occupational class men (professionals and managers) had significantly lower odds ratios for stomach and lung cancer, with the exception of risk for stomach cancer in managers in blue‐collar industries (Table 1). Even after fully controlling for smoking and alcohol consumption, the observed lower odds in higher occupational class across all industries were not attenuated and remained significantly associated with stomach cancer (adjusted OR ranged from 0.80 for managers in white‐collar industries to 0.93 for professionals in blue‐collar industries) and lung cancer (adjusted OR ranged from 0.66 for managers in white‐collar industries to 0.83 for managers in blue‐collar industries; model 2, Table 1). Additionally, service workers in all industries and blue‐collar workers in service and white‐collar industries also had significantly lower odds ratios for lung cancer.

**Table 1:** Odds ratios of each occupational class associated with risk for top 10 common cancers and overall cancer incidence in Japan

Characteristics	Control, %^a^	Case, %^a^	Model 1 OR (95%CI)^b^	Model 2 OR (95%CI)^b^
*Esophagus*	n = 30 545	n = 6317	OR (95% CI)^b^	OR (95% CI)^b^
Occupational class				
Blue‐collar industry				
Blue‐collar	32.4	34.5	1.00	1.00
Service	11.2	11.4	0.96 (0.87‐1.06)	0.95 (0.85‐1.05)
Professional	3.3	2.9	0.82 (0.67‐0.99)	0.81 (0.66‐0.98)
Manager	4.3	4.6	0.99 (0.85‐1.16)	0.95 (0.81‐1.11)
Service industry				
Blue‐collar	3.0	3.1	1.00 (0.83‐1.21)	1.03 (0.84‐1.24)
Service	10.6	11.2	1.01 (0.91‐1.11)	1.02 (0.92‐1.13)
Professional	0.9	1.0	1.04 (0.77‐1.40)	1.02 (0.75‐1.40)
Manager	2.2	2.1	0.91 (0.74‐1.13)	0.90 (0.72‐1.13)
White‐collar industry				
Blue‐collar	1.9	2.0	1.00 (0.81‐1.25)	1.03 (0.83‐1.27)
Service	6.7	5.9	0.83 (0.71‐0.95)	0.82 (0.71‐0.95)
Professional	4.8	4.0	0.78 (0.66‐0.93)	0.82 (0.70‐0.97)
Manager	1.4	1.1	0.70 (0.49‐0.99)	0.73 (0.52‐1.02)
Others				
Others	17.3	16.2	0.86 (0.78‐0.94)	0.96 (0.87‐1.06)
Smoking, mean^c^	2.31	2.86		1.19 (1.16‐1.21)
Alcohol consumption, mean^d^	2.37	3.02		1.29 (1.26‐1.33)
*Stomach*	n = 203 506	n = 42 510		
Occupational class				
Blue‐collar industry				
Blue‐collar	32.5	35.3	1.00	1.00
Service	10.8	11.0	0.95 (0.90‐0.99)	0.94 (0.90‐0.99)
Professional	3.0	3.0	0.93 (0.87‐0.99)	0.93 (0.87‐1.00)
Manager	4.3	4.4	0.95 (0.90‐1.02)	0.93 (0.87‐0.99)
Service industry				
Blue‐collar	2.9	3.0	0.94 (0.86‐1.01)	0.94 (0.87‐1.02)
Service	10.6	10.3	0.91 (0.87‐0.95)	0.91 (0.87‐0.95)
Professional	0.9	0.8	0.85 (0.73‐0.98)	0.86 (0.74‐1.00)
Manager	2.2	2.0	0.86 (0.79‐0.94)	0.86 (0.79‐0.94)
White‐collar industry				
Blue‐collar	1.9	1.9	0.92 (0.84‐1.01)	0.93 (0.85‐1.02)
Service	6.9	6.3	0.84 (0.80‐0.89)	0.85 (0.81‐0.90)
Professional	5.0	4.2	0.77 (0.72‐0.82)	0.80 (0.75‐0.86)
Manager	1.5	1.3	0.79 (0.71‐0.89)	0.80 (0.72‐0.90)
Others				
Others	17.8	16.5	0.83 (0.80‐0.86)	0.86 (0.83‐0.89)
Smoking, mean^c^	2.26	2.59		1.12 (1.11‐1.13)
Alcohol consumption, mean^d^	2.32	2.53		1.06 (1.05‐1.07)
*Colorectum*	n = 128 696	n = 27 074		
Occupational class				
Blue‐collar industry				
Blue‐collar	31.6	32.3	1.00	1.00
Service	11.5	11.7	1.01 (0.96‐1.07)	1.01 (0.96‐1.07)
Professional	3.4	3.5	1.02 (0.94‐1.12)	1.02 (0.94‐1.12)
Manager	4.1	4.0	0.99 (0.92‐1.06)	0.97 (0.90‐1.04)
Service industry				
Blue‐collar	3.0	3.2	1.05 (0.97‐1.14)	1.07 (0.98‐1.15)
Service	11.0	11.4	1.02 (0.96‐1.08)	1.02 (0.96‐1.08)
Professional	1.0	0.9	0.91 (0.77‐1.09)	0.93 (0.78‐1.10)
Manager	2.0	2.1	1.01 (0.91‐1.13)	1.01 (0.90‐1.13)
White‐collar industry				
Blue‐collar	1.9	1.8	0.89 (0.77‐1.02)	0.89 (0.77‐1.02)
Service	7.1	6.9	0.96 (0.89‐1.04)	0.97 (0.90‐1.04)
Professional	5.1	5.1	0.96 (0.89‐1.04)	0.99 (0.92‐1.06)
Manager	1.4	1.2	0.88 (0.77‐0.99)	0.88 (0.78‐1.00)
Others				
Others	17.0	16.1	0.90 (0.85‐0.95)	0.94 (0.89‐0.99)
Smoking, mean^c^	2.38	2.56		1.06 (1.05‐1.07)
Alcohol consumption, mean^d^	2.45	2.67		1.09 (1.08‐1.10)
*Liver*	n = 88 342	n = 18 354		
Occupational class				
Blue‐collar industry				
Blue‐collar	31.9	32.7	1.00	1.00
Service	11.1	11.6	1.02 (0.96‐1.08)	1.02 (0.96‐1.08)
Professional	3.1	2.8	0.87 (0.76‐0.99)	0.87 (0.76‐0.99)
Manager	4.6	5.1	1.09 (1.00‐1.19)	1.07 (0.98‐1.17)
Service industry				
Blue‐collar	2.9	3.1	1.03 (0.93‐1.14)	1.04 (0.94‐1.15)
Service	10.7	10.6	0.97 (0.91‐1.03)	0.97 (0.92‐1.03)
Professional	0.8	0.7	0.89 (0.73‐1.09)	0.91 (0.75‐1.11)
Manager	2.1	2.2	1.01 (0.88‐1.16)	1.01 (0.88‐1.16)
White‐collar industry				
Blue‐collar	1.9	1.7	0.84 (0.74‐0.96)	0.84 (0.74‐0.96)
Service	7.0	6.0	0.84 (0.77‐0.92)	0.85 (0.78‐0.93)
Professional	4.9	3.7	0.74 (0.67‐0.81)	0.76 (0.69‐0.84)
Manager	1.6	1.3	0.81 (0.67‐0.97)	0.81 (0.68‐0.97)
Others				
Others	17.3	18.6	1.04 (0.98‐1.10)	1.07 (1.00‐1.14)
Smoking, mean^c^	2.28	2.51		1.09 (1.07‐1.10)
Alcohol consumption, mean^d^	2.34	2.49		1.04 (1.02‐1.05)
*Pancreas*	n = 23 635	n = 4976		
Occupational class				
Blue‐collar industry				
Blue‐collar	31.9	33.6	1.00	1.00
Service	10.7	11.7	1.04 (0.93‐1.16)	1.03 (0.93‐1.15)
Professional	3.1	2.9	0.88 (0.69‐1.13)	0.89 (0.70‐1.13)
Manager	4.4	4.4	0.96 (0.80‐1.16)	0.95 (0.79‐1.14)
Service industry				
Blue‐collar	3.1	3.2	0.99 (0.80‐1.22)	1.01 (0.82‐1.24)
Service	10.5	10.2	0.92 (0.77‐1.11)	0.93 (0.77‐1.12)
Professional	0.9	0.9	0.92 (0.62‐1.39)	0.93 (0.62‐1.40)
Manager	2.1	2.2	1.00 (0.79‐1.27)	1.00 (0.79‐1.27)
White‐collar industry				
Blue‐collar	2.0	1.6	0.75 (0.58‐0.98)	0.76 (0.58‐0.99)
Service	6.7	5.9	0.83 (0.72‐0.96)	0.84 (0.73‐0.96)
Professional	4.8	4.5	0.90 (0.75‐1.07)	0.93 (0.78‐1.11)
Manager	1.5	1.3	0.83 (0.62‐1.11)	0.85 (0.63‐1.14)
Others				
Others	18.2	17.6	0.88 (0.80‐0.97)	0.91 (0.83‐1.01)
Smoking, mean^c^	2.28	2.61		1.14 (1.11‐1.17)
Alcohol consumption, mean^d^	2.33	2.41		1.00 (0.98‐1.03)
*Lung*	n = 104 064	n = 21 922		
Occupational class				
Blue‐collar industry				
Blue‐collar	32.6	37.5	1.00	1.00
Service	10.6	10.6	0.87 (0.83‐0.93)	0.86 (0.82‐0.91)
Professional	3.1	2.7	0.75 (0.68‐0.84)	0.76 (0.68‐0.85)
Manager	4.0	3.9	0.86 (0.79‐0.93)	0.83 (0.76‐0.90)
Service industry				
Blue‐collar	2.8	2.9	0.89 (0.81‐0.98)	0.89 (0.81‐0.98)
Service	10.0	9.4	0.82 (0.77‐0.87)	0.83 (0.78‐0.89)
Professional	0.9	0.7	0.65 (0.54‐0.77)	0.68 (0.56‐0.82)
Manager	2.0	1.9	0.80 (0.71‐0.90)	0.81 (0.72‐0.92)
White‐collar industry				
Blue‐collar	1.7	1.5	0.76 (0.66‐0.88)	0.79 (0.69‐0.91)
Service	6.3	5.4	0.75 (0.68‐0.82)	0.77 (0.70‐0.84)
Professional	4.6	3.2	0.61 (0.55‐0.66)	0.66 (0.60‐0.73)
Manager	1.4	1.0	0.61 (0.51‐0.72)	0.66 (0.55‐0.79)
Others				
Others	19.8	19.2	0.82 (0.79‐0.86)	0.90 (0.86‐0.95)
Smoking, mean^c^	2.33	3.04		1.36 (1.35‐1.38)
Alcohol consumption, mean^d^	2.31	2.43		0.99 (0.98‐1.00)
*Prostate*	n = 136 573	n = 28 392		
Occupational class				
Blue‐collar industry				
Blue‐collar	31.5	31.8	1.00	1.00
Service	11.4	12.0	1.06 (1.01‐1.12)	1.06 (1.01‐1.12)
Professional	3.5	3.6	1.06 (0.99‐1.15)	1.06 (0.98‐1.14)
Manager	3.9	3.9	1.02 (0.94‐1.10)	1.02 (0.94‐1.10)
Service industry				
Blue‐collar	3.0	2.7	0.90 (0.82‐0.99)	0.91 (0.83‐0.99)
Service	10.4	10.1	0.97 (0.91‐1.03)	0.97 (0.91‐1.03)
Professional	1.1	1.1	0.98 (0.86‐1.11)	0.98 (0.86‐1.11)
Manager	2.1	2.0	0.96 (0.86‐1.06)	0.96 (0.86‐1.06)
White‐collar industry				
Blue‐collar	1.9	2.0	1.07 (0.96‐1.20)	1.07 (0.95‐1.19)
Service	6.5	6.7	1.03 (0.97‐1.10)	1.03 (0.97‐1.10)
Professional	4.9	5.4	1.10 (1.03‐1.18)	1.10 (1.03‐1.18)
Manager	1.2	1.3	1.07 (0.94‐1.22)	1.07 (0.93‐1.22)
Others				
Others	18.5	17.3	0.90 (0.86‐0.94)	0.90 (0.86‐0.94)
Smoking, mean^c^	2.41	2.37		0.98 (0.97‐0.99)
Alcohol consumption, mean^d^	2.36	2.43		1.03 (1.02‐1.05)
*Kidney, pelvis and ureter*	n = 26 900	n = 5552		
Occupational class				
Blue‐collar industry				
Blue‐collar	31.4	31.4	1.00	1.00
Service	11.9	12.1	1.03 (0.93‐1.14)	1.03 (0.93‐1.14)
Professional	3.8	3.8	1.04 (0.81‐1.35)	1.05 (0.81‐1.36)
Manager	4.0	4.7	1.19 (1.02‐1.39)	1.17 (1.00‐1.37)
Service industry				
Blue‐collar	2.9	3.1	1.07 (0.87‐1.32)	1.08 (0.87‐1.33)
Service	11.0	10.8	0.99 (0.88‐1.11)	0.99 (0.88‐1.11)
Professional	0.9	1.0	1.17 (0.81‐1.67)	1.17 (0.82‐1.67)
Manager	2.0	2.3	1.15 (0.93‐1.42)	1.15 (0.92‐1.42)
White‐collar industry				
Blue‐collar	2.1	1.7	0.84 (0.65‐1.09)	0.84 (0.65‐1.10)
Service	7.2	7.3	1.02 (0.88‐1.17)	1.03 (0.89‐1.18)
Professional	5.3	5.4	1.04 (0.88‐1.22)	1.07 (0.90‐1.26)
Manager	1.4	1.4	0.97 (0.72‐1.29)	0.97 (0.73‐1.30)
Others				
Others	16.1	15.1	0.93 (0.82‐1.04)	0.95 (0.85‐1.07)
Smoking, mean^c^	2.35	2.58		1.08 (1.06‐1.11)
Alcohol consumption, mean^d^	2.41	2.58		1.05 (1.03‐1.08)
*Bladder*	n = 64 871	n = 13 590		
Occupational class				
Blue‐collar industry				
Blue‐collar	31.3	32.8	1.00	1.00
Service	10.6	11.6	1.06 (0.98‐1.15)	1.05 (0.97‐1.14)
Professional	3.2	3.0	0.91 (0.80‐1.03)	0.90 (0.79‐1.03)
Manager	4.3	4.6	1.05 (0.95‐1.16)	1.02 (0.92‐1.13)
Service industry				
Blue‐collar	2.9	2.7	0.90 (0.79‐1.03)	0.90 (0.78‐1.03)
Service	10.1	10.4	0.99 (0.93‐1.06)	1.00 (0.93‐1.07)
Professional	0.9	1.0	1.14 (0.93‐1.39)	1.14 (0.92‐1.40)
Manager	2.1	2.2	1.02 (0.88‐1.18)	1.02 (0.88‐1.19)
White‐collar industry				
Blue‐collar	1.8	1.6	0.89 (0.76‐1.03)	0.89 (0.77‐1.04)
Service	6.7	5.9	0.84 (0.75‐0.95)	0.85 (0.76‐0.95)
Professional	4.9	4.5	0.88 (0.78‐0.98)	0.92 (0.82‐1.02)
Manager	1.4	1.2	0.78 (0.62‐0.98)	0.78 (0.63‐0.98)
Others				
Others	19.9	18.4	0.86 (0.81‐0.91)	0.89 (0.84‐0.94)
Smoking, mean^c^	2.29	2.69		1.17 (1.15‐1.18)
Alcohol consumption, mean^d^	2.31	2.43		1.02 (1.00‐1.03)
*Malignant lymphoma*	n = 29 528	n = 6157		
Occupational class				
Blue‐collar industry				
Blue‐collar	31.0	33.4	1.00	1.00
Service	11.7	11.5	0.92 (0.83‐1.02)	0.92 (0.83‐1.01)
Professional	3.8	3.4	0.82 (0.69‐0.96)	0.82 (0.70‐0.97)
Manager	3.8	3.9	0.96 (0.76‐1.21)	0.95 (0.75‐1.20)
Service industry				
Blue‐collar	3.1	3.8	1.14 (0.97‐1.34)	1.14 (0.97‐1.33)
Service	11.0	10.1	0.86 (0.77‐0.96)	0.86 (0.77‐0.96)
Professional	0.9	1.0	0.94 (0.68‐1.30)	0.94 (0.69‐1.30)
Manager	1.9	1.9	0.92 (0.69‐1.22)	0.92 (0.69‐1.21)
White‐collar industry				
Blue‐collar	2.0	1.8	0.82 (0.65‐1.04)	0.83 (0.65‐1.04)
Service	7.5	6.9	0.86 (0.75‐0.98)	0.86 (0.76‐0.98)
Professional	5.5	5.1	0.85 (0.72‐1.01)	0.87 (0.73‐1.03)
Manager	1.4	1.2	0.85 (0.60‐1.19)	0.85 (0.61‐1.20)
Others				
Others	16.4	16.2	0.90 (0.82‐0.99)	0.91 (0.83‐1.00)
Smoking, mean^c^	2.30	2.44		1.06 (1.03‐1.09)
Alcohol consumption, mean^d^	2.39	2.40		0.99 (0.97‐1.02)
*All sites*	n = 1 026 247	n = 214 123		
Occupational class				
Blue‐collar industry				
Blue‐collar	31.8	33.6	1.00	1.00
Service	11.1	11.4	0.99 (0.97‐1.00)	0.98 (0.96‐1.00)
Professional	3.3	3.1	0.92 (0.88‐0.96)	0.92 (0.88‐0.96)
Manager	4.2	4.3	0.98 (0.96‐1.01)	0.97 (0.94‐0.99)
Service industry				
Blue‐collar	2.9	3.0	0.97 (0.94‐1.00)	0.97 (0.94‐1.00)
Service	10.6	10.6	0.95 (0.93‐0.96)	0.95 (0.94‐0.97)
Professional	0.9	0.9	0.92 (0.86‐0.98)	0.93 (0.87‐1.00)
Manager	2.1	2.0	0.93 (0.89‐0.97)	0.93 (0.89‐0.97)
White‐collar industry				
Blue‐collar	1.9	1.8	0.90 (0.86‐0.94)	0.90 (0.86‐0.95)
Service	6.9	6.3	0.88 (0.86‐0.90)	0.89 (0.86‐0.91)
Professional	5.0	4.5	0.86 (0.83‐0.88)	0.89 (0.86‐0.92)
Manager	1.4	1.2	0.82 (0.78‐0.86)	0.83 (0.79‐0.87)
Others				
Others	17.9	17.3	0.89 (0.88‐0.91)	0.92 (0.91‐0.94)
Smoking, mean^c^	2.31	2.58		1.10 (1.10‐1.11)
Alcohol consumption, mean^d^	2.35	2.51		1.05 (1.04‐1.05)

CI, confidence interval; OR, odds ratio.

a Data were estimated with five imputed datasets. Percentages may not total 100 because of rounding with multiple imputation.

b Conditional logistic regression with multiple imputation, matched for age, admission date, and admitting hospital (model 1); additional adjustment for smoking and alcohol consumption (model 2).

c Log (1 + pack‐year).

d Log (1 + daily gram of ethanol intake).

Among the remainder of the top 10 common cancers, higher occupational class in white‐collar industries was associated with reduced risks for liver, esophagus, and bladder cancer, as well as malignant lymphoma (Table 1). Higher occupational class tended to be associated with potentially lower risk for pancreatic cancer (although not statistically significant), while occupational class was not clearly associated with colorectal cancer risk (Table 1). By contrast, an excess cancer risk was associated with professionals in white‐collar industries for prostate cancer, as well as a tendency of excess risk with higher occupational class in blue‐collar industries was observed for kidney cancer (Table 1). As a whole, a reduced risk was associated with higher occupational class for overall cancer incidence (Table 1).

Some less common cancers (such as gallbladder and bile duct cancer, leukemia, and multiple myeloma) appeared to hint at a reduced risk with higher occupational class (Table S2). The results of sensitivity analyses showed almost the same occupational gradient patterns as seen in the main result (Tables 2 and 3; Table S3 and Figure S3).

**Table 2:** Odds ratios of each occupational class associated with risk for stomach, lung, prostate, and overall cancer incidence stratified by age

Occupational class	Control, %^a^	Case, %^a^	Model 1 OR (95%CI)^b^	Model 2 OR (95%CI)^b^
*Stomach*				
Age 20‐64	n = 82 294	n = 16 925	OR (95% CI)^b^	OR (95% CI)^b^
Blue‐collar industry				
Blue‐collar	32.6	35.9	1.00	1.00
Service	12.4	12.6	0.97 (0.92‐1.02)	0.96 (0.91‐1.02)
Professional	3.7	3.8	0.93 (0.83‐1.04)	0.93 (0.83‐1.04)
Manager	4.6	4.9	0.95 (0.87‐1.02)	0.93 (0.86‐1.00)
Service industry				
Blue‐collar	3.6	3.5	0.99 (0.89‐1.10)	0.99 (0.89‐1.10)
Service	12.9	12.9	0.90 (0.85‐0.95)	0.90 (0.85‐0.96)
Professional	0.7	0.6	0.90 (0.76‐1.06)	0.91 (0.77‐1.08)
Manager	2.1	2.0	0.86 (0.77‐0.96)	0.87 (0.77‐0.97)
White‐collar industry				
Blue‐collar	2.3	2.4	0.88 (0.79‐0.99)	0.90 (0.81‐1.01)
Service	8.8	8.2	0.84 (0.78‐0.91)	0.84 (0.78‐0.91)
Professional	5.7	4.7	0.79 (0.73‐0.85)	0.82 (0.76‐0.88)
Manager	1.6	1.5	0.75 (0.65‐0.87)	0.76 (0.66‐0.88)
Others				
Others	9.0	6.9	0.87 (0.83‐0.91)	0.90 (0.86‐0.94)
Age 65 and above	n = 121 212	n = 25 585		
Blue‐collar industry				
Blue‐collar	32.4	35.0	1.00	1.00
Service	9.6	10.0	0.92 (0.86‐0.99)	0.91 (0.85‐0.98)
Professional	2.5	2.5	0.92 (0.84‐1.01)	0.93 (0.84‐1.02)
Manager	4.0	4.1	0.96 (0.88‐1.05)	0.93 (0.85‐1.01)
Service industry				
Blue‐collar	2.4	2.6	0.88 (0.79‐0.99)	0.89 (0.80‐1.00)
Service	9.0	8.6	0.91 (0.86‐0.97)	0.91 (0.86‐0.97)
Professional	0.9	0.9	0.76 (0.58‐0.99)	0.78 (0.59‐1.02)
Manager	2.2	2.0	0.86 (0.76‐0.99)	0.85 (0.74‐0.97)
White‐collar industry				
Blue‐collar	1.7	1.6	0.95 (0.82‐1.11)	0.96 (0.82‐1.11)
Service	5.6	5.1	0.84 (0.78‐0.90)	0.86 (0.80‐0.92)
Professional	4.4	3.8	0.74 (0.67‐0.83)	0.78 (0.70‐0.87)
Manager	1.4	1.1	0.84 (0.73‐0.98)	0.85 (0.73‐0.99)
Others				
Others	23.8	22.8	0.69 (0.63‐0.75)	0.73 (0.67‐0.80)
*Lung*				
Age 20‐64	n = 28 411	n = 5893		
Blue‐collar industry				
Blue‐collar	32.6	35.9	1.00	1.00
Service	12.4	12.6	0.90 (0.84‐0.97)	0.89 (0.83‐0.95)
Professional	3.7	3.8	0.81 (0.72‐0.92)	0.81 (0.72‐0.91)
Manager	4.6	4.9	0.89 (0.80‐0.98)	0.86 (0.77‐0.95)
Service industry				
Blue‐collar	3.6	3.5	0.93 (0.83‐1.05)	0.92 (0.82‐1.03)
Service	12.9	12.9	0.83 (0.76‐0.89)	0.84 (0.77‐0.91)
Professional	0.7	0.6	0.63 (0.51‐0.78)	0.65 (0.52‐0.82)
Manager	2.1	2.0	0.81 (0.70‐0.93)	0.82 (0.71‐0.96)
White‐collar industry				
Blue‐collar	2.3	2.4	0.81 (0.68‐0.96)	0.85 (0.71‐1.02)
Service	8.8	8.2	0.75 (0.68‐0.84)	0.76 (0.69‐0.85)
Professional	5.7	4.7	0.62 (0.55‐0.70)	0.68 (0.60‐0.76)
Manager	1.6	1.5	0.65 (0.52‐0.81)	0.70 (0.56‐0.89)
Others				
Others	9.0	6.9	0.84 (0.80‐0.88)	0.92 (0.87‐0.97)
Age 65 and above	n = 75 653	n = 16 029		
Blue‐collar industry				
Blue‐collar	32.4	35.0	1.00	1.00
Service	9.6	10.0	0.82 (0.73‐0.91)	0.81 (0.72‐0.91)
Professional	2.5	2.5	0.65 (0.54‐0.78)	0.67 (0.55‐0.80)
Manager	4.0	4.1	0.78 (0.66‐0.94)	0.76 (0.64‐0.91)
Service industry				
Blue‐collar	2.4	2.6	0.80 (0.68‐0.94)	0.82 (0.70‐0.97)
Service	9.0	8.6	0.80 (0.72‐0.88)	0.80 (0.72‐0.88)
Professional	0.9	0.9	0.71 (0.48‐1.03)	0.76 (0.52‐1.12)
Manager	2.2	2.0	0.78 (0.63‐0.97)	0.78 (0.62‐0.98)
White‐collar industry				
Blue‐collar	1.7	1.6	0.67 (0.51‐0.87)	0.67 (0.51‐0.88)
Service	5.6	5.1	0.74 (0.65‐0.84)	0.77 (0.67‐0.87)
Professional	4.4	3.8	0.57 (0.48‐0.67)	0.63 (0.53‐0.74)
Manager	1.4	1.1	0.52 (0.34‐0.80)	0.55 (0.36‐0.85)
Others				
Others	23.8	22.8	0.77 (0.69‐0.87)	0.86 (0.76‐0.97)
*Prostate*				
Age 20‐64	n = 25 068	n = 5117		
Blue‐collar industry				
Blue‐collar	32.6	35.9	1.00	1.00
Service	12.4	12.6	1.06 (1.00‐1.13)	1.06 (1.00‐1.12)
Professional	3.7	3.8	1.00 (0.92‐1.10)	1.00 (0.91‐1.10)
Manager	4.6	4.9	1.01 (0.92‐1.10)	1.00 (0.92‐1.10)
Service industry				
Blue‐collar	3.6	3.5	0.92 (0.83‐1.02)	0.92 (0.83‐1.03)
Service	12.9	12.9	0.97 (0.91‐1.03)	0.97 (0.91‐1.03)
Professional	0.7	0.6	0.99 (0.86‐1.14)	0.99 (0.87‐1.14)
Manager	2.1	2.0	0.90 (0.81‐1.01)	0.90 (0.81‐1.01)
White‐collar industry				
Blue‐collar	2.3	2.4	1.02 (0.90‐1.16)	1.02 (0.90‐1.15)
Service	8.8	8.2	0.97 (0.90‐1.05)	0.97 (0.90‐1.05)
Professional	5.7	4.7	1.03 (0.96‐1.11)	1.03 (0.96‐1.11)
Manager	1.6	1.5	1.03 (0.89‐1.19)	1.03 (0.89‐1.19)
Others				
Others	9.0	6.9	0.91 (0.86‐0.95)	0.91 (0.86‐0.95)
Age 65 and above	n = 111 505	n = 23 275		
Blue‐collar industry				
Blue‐collar	32.4	35.0	1.00	1.00
Service	9.6	10.0	1.07 (0.96‐1.19)	1.08 (0.96‐1.20)
Professional	2.5	2.5	1.27 (1.09‐1.47)	1.26 (1.08‐1.47)
Manager	4.0	4.1	1.07 (0.91‐1.26)	1.07 (0.91‐1.26)
Service industry				
Blue‐collar	2.4	2.6	0.86 (0.71‐1.04)	0.86 (0.70‐1.04)
Service	9.0	8.6	1.00 (0.90‐1.11)	1.01 (0.90‐1.12)
Professional	0.9	0.9	0.88 (0.56‐1.38)	0.87 (0.56‐1.37)
Manager	2.2	2.0	1.22 (0.99‐1.51)	1.23 (0.99‐1.52)
White‐collar industry				
Blue‐collar	1.7	1.6	1.26 (1.03‐1.55)	1.26 (1.03‐1.54)
Service	5.6	5.1	1.25 (1.12‐1.40)	1.25 (1.11‐1.40)
Professional	4.4	3.8	1.41 (1.22‐1.62)	1.39 (1.21‐1.60)
Manager	1.4	1.1	1.25 (0.96‐1.62)	1.24 (0.95‐1.61)
Others				
Others	23.8	22.8	0.78 (0.68‐0.89)	0.78 (0.68‐0.89)
*All sites*				
Age 20‐64	n = 374 853	n = 77 173		
Blue‐collar industry				
Blue‐collar	31.6	33.4	1.00	1.00
Service	12.8	13.1	1.00 (0.98‐1.02)	0.99 (0.97‐1.02)
Professional	4.2	4.0	0.93 (0.89‐0.98)	0.93 (0.89‐0.97)
Manager	4.5	4.5	0.99 (0.96‐1.03)	0.97 (0.94‐1.01)
Service industry				
Blue‐collar	3.6	3.7	0.98 (0.93‐1.02)	0.98 (0.93‐1.02)
Service	13.1	13.3	0.94 (0.92‐0.96)	0.95 (0.93‐0.97)
Professional	0.7	0.8	0.90 (0.84‐0.96)	0.91 (0.85‐0.98)
Manager	2.1	2.1	0.92 (0.87‐0.97)	0.92 (0.87‐0.97)
White‐collar industry				
Blue‐collar	2.3	2.2	0.90 (0.85‐0.95)	0.90 (0.85‐0.96)
Service	8.9	8.4	0.86 (0.84‐0.89)	0.87 (0.84‐0.89)
Professional	5.9	5.3	0.87 (0.83‐0.90)	0.89 (0.86‐0.93)
Manager	1.6	1.4	0.80 (0.74‐0.87)	0.81 (0.75‐0.88)
Others				
Others	8.7	8.0	0.90 (0.88‐0.92)	0.93 (0.91‐0.95)
Age 65 and above	n = 651 394	n = 136 950		
Blue‐collar industry				
Blue‐collar	32.0	33.7	1.00	1.00
Service	10.1	10.5	0.97 (0.94‐1.00)	0.96 (0.93‐0.99)
Professional	2.8	2.7	0.90 (0.85‐0.96)	0.91 (0.85‐0.97)
Manager	4.0	4.1	0.97 (0.92‐1.02)	0.95 (0.90‐1.00)
Service industry				
Blue‐collar	2.5	2.6	0.95 (0.91‐1.00)	0.97 (0.92‐1.01)
Service	9.2	9.0	0.96 (0.93‐0.98)	0.96 (0.94‐0.99)
Professional	1.0	0.9	0.96 (0.86‐1.07)	0.98 (0.88‐1.10)
Manager	2.1	2.0	0.95 (0.90‐1.01)	0.95 (0.89‐1.01)
White‐collar industry				
Blue‐collar	1.7	1.6	0.90 (0.84‐0.96)	0.90 (0.84‐0.97)
Service	5.7	5.1	0.90 (0.86‐0.94)	0.91 (0.87‐0.96)
Professional	4.5	4.1	0.85 (0.81‐0.89)	0.88 (0.85‐0.92)
Manager	1.3	1.1	0.84 (0.78‐0.91)	0.84 (0.78‐0.91)
Others				
Others	23.2	22.5	0.86 (0.83‐0.90)	0.91 (0.87‐0.95)

CI, confidence interval; OR, odds ratio.

a Data were estimated with five imputed datasets. Percentages may not total 100 because of rounding with multiple imputation.

b Conditional logistic regression with multiple imputation, matched for age, admission date, and admitting hospital (model 1); additional adjustment for smoking and alcohol consumption (model 2).

**Table 3:** Odds ratios of each occupational class associated with risk for stomach, lung, prostate, and overall cancer incidence stratified by admission date

Occupational class	Control, %^a^	Case, %^a^	Model 1 OR (95%CI)^b^	Model 2 OR (95%CI)^b^
*Stomach*				
Before 2003	n = 120 886	n = 25 081	OR (95% CI)^b^	OR (95% CI)^b^
Blue‐collar industry				
Blue‐collar	33.4	35.8	1.00	1.00
Service	9.8	9.9	0.94 (0.89‐1.00)	0.93 (0.88‐0.99)
Professional	2.4	2.4	0.91 (0.82‐1.00)	0.90 (0.82‐1.00)
Manager	4.7	5.0	0.88 (0.79‐0.98)	0.85 (0.76‐0.94)
Service industry				
Blue‐collar	2.7	2.6	0.95 (0.86‐1.05)	0.96 (0.86‐1.06)
Service	9.9	9.8	0.88 (0.83‐0.94)	0.88 (0.82‐0.94)
Professional	0.6	0.6	0.81 (0.67‐0.97)	0.83 (0.68‐1.00)
Manager	2.3	2.3	0.77 (0.65‐0.92)	0.76 (0.63‐0.90)
White‐collar industry				
Blue‐collar	1.9	1.8	0.93 (0.81‐1.05)	0.93 (0.81‐1.06)
Service	6.7	6.0	0.85 (0.78‐0.93)	0.85 (0.78‐0.93)
Professional	4.7	4.1	0.72 (0.66‐0.79)	0.76 (0.69‐0.83)
Manager	1.7	1.6	0.66 (0.53‐0.81)	0.67 (0.54‐0.82)
Others				
Others	19.2	18.2	0.77 (0.72‐0.83)	0.82 (0.77‐0.87)
After 2003	n = 82 620	n = 17 429		
Blue‐collar industry				
Blue‐collar	31.1	34.7	1.00	1.00
Service	12.2	12.7	0.95 (0.87‐1.03)	0.94 (0.87‐1.02)
Professional	3.8	3.8	0.94 (0.85‐1.04)	0.95 (0.86‐1.04)
Manager	3.7	3.5	1.00 (0.93‐1.08)	0.98 (0.91‐1.05)
Service industry				
Blue‐collar	3.2	3.4	0.92 (0.81‐1.04)	0.93 (0.82‐1.05)
Service	11.5	11.2	0.92 (0.87‐0.97)	0.93 (0.88‐0.98)
Professional	1.2	1.0	0.89 (0.71‐1.10)	0.90 (0.72‐1.13)
Manager	2.0	1.7	0.92 (0.82‐1.02)	0.92 (0.83‐1.03)
White‐collar industry				
Blue‐collar	2.0	2.0	0.91 (0.78‐1.06)	0.93 (0.80‐1.08)
Service	7.2	6.8	0.84 (0.78‐0.90)	0.85 (0.79‐0.91)
Professional	5.3	4.3	0.81 (0.74‐0.88)	0.84 (0.77‐0.91)
Manager	1.2	0.9	0.87 (0.77‐0.98)	0.87 (0.78‐0.99)
Others				
Others	15.7	14.0	0.86 (0.82‐0.91)	0.89 (0.84‐0.94)
*Lung*				
Before 2003	n = 50 718	n = 10 614		
Blue‐collar industry				
Blue‐collar	33.4	35.8	1.00	1.00
Service	9.8	9.9	0.87 (0.82‐0.94)	0.85 (0.80‐0.92)
Professional	2.4	2.4	0.72 (0.62‐0.84)	0.72 (0.62‐0.85)
Manager	4.7	5.0	0.81 (0.70‐0.92)	0.76 (0.66‐0.88)
Service industry				
Blue‐collar	2.7	2.6	0.87 (0.77‐0.99)	0.86 (0.75‐0.98)
Service	9.9	9.8	0.79 (0.72‐0.87)	0.79 (0.72‐0.87)
Professional	0.6	0.6	0.59 (0.47‐0.74)	0.62 (0.49‐0.78)
Manager	2.3	2.3	0.65 (0.54‐0.77)	0.63 (0.53‐0.76)
White‐collar industry				
Blue‐collar	1.9	1.8	0.71 (0.59‐0.85)	0.72 (0.60‐0.88)
Service	6.7	6.0	0.74 (0.66‐0.83)	0.75 (0.67‐0.85)
Professional	4.7	4.1	0.54 (0.47‐0.62)	0.61 (0.52‐0.71)
Manager	1.7	1.6	0.55 (0.42‐0.70)	0.62 (0.48‐0.80)
Others				
Others	19.2	18.2	0.73 (0.68‐0.79)	0.82 (0.76‐0.89)
After 2003	n = 53 346	n = 11 308		
Blue‐collar industry				
Blue‐collar	31.1	34.7	1.00	1.00
Service	12.2	12.7	0.86 (0.78‐0.96)	0.86 (0.77‐0.95)
Professional	3.8	3.8	0.80 (0.67‐0.95)	0.80 (0.67‐0.96)
Manager	3.7	3.5	0.91 (0.81‐1.01)	0.89 (0.80‐1.00)
Service industry				
Blue‐collar	3.2	3.4	0.90 (0.76‐1.06)	0.91 (0.77‐1.07)
Service	11.5	11.2	0.85 (0.77‐0.93)	0.87 (0.79‐0.95)
Professional	1.2	1.0	0.74 (0.55‐0.99)	0.77 (0.57‐1.03)
Manager	2.0	1.7	0.96 (0.82‐1.13)	1.00 (0.85‐1.17)
White‐collar industry				
Blue‐collar	2.0	2.0	0.82 (0.67‐1.01)	0.86 (0.70‐1.05)
Service	7.2	6.8	0.76 (0.67‐0.86)	0.78 (0.69‐0.88)
Professional	5.3	4.3	0.69 (0.60‐0.79)	0.74 (0.64‐0.84)
Manager	1.2	0.9	0.66 (0.53‐0.83)	0.70 (0.55‐0.88)
Others				
Others	15.7	14.0	0.92 (0.86‐0.98)	0.98 (0.92‐1.05)
*Prostate*				
Before 2003	n = 40 290	n = 8444		
Blue‐collar industry				
Blue‐collar	33.4	35.8	1.00	1.00
Service	9.8	9.9	1.06 (1.00‐1.12)	1.06 (1.00‐1.12)
Professional	2.4	2.4	1.12 (1.03‐1.22)	1.12 (1.03‐1.21)
Manager	4.7	5.0	1.02 (0.93‐1.12)	1.02 (0.93‐1.12)
Service industry				
Blue‐collar	2.7	2.6	0.89 (0.80‐1.00)	0.89 (0.80‐1.00)
Service	9.9	9.8	0.97 (0.90‐1.04)	0.97 (0.90‐1.04)
Professional	0.6	0.6	0.91 (0.78‐1.05)	0.91 (0.78‐1.05)
Manager	2.3	2.3	0.88 (0.77‐1.00)	0.88 (0.77‐1.00)
White‐collar industry				
Blue‐collar	1.9	1.8	1.07 (0.92‐1.24)	1.06 (0.91‐1.23)
Service	6.7	6.0	1.07 (0.99‐1.15)	1.06 (0.99‐1.14)
Professional	4.7	4.1	1.09 (1.01‐1.18)	1.08 (1.00‐1.17)
Manager	1.7	1.6	1.12 (0.95‐1.32)	1.12 (0.95‐1.32)
Others				
Others	19.2	18.2	0.88 (0.83‐0.93)	0.88 (0.83‐0.93)
After 2003	n = 96 283	n = 19 948		
Blue‐collar industry				
Blue‐collar	31.1	34.7	1.00	1.00
Service	12.2	12.7	1.06 (0.97‐1.17)	1.06 (0.97‐1.17)
Professional	3.8	3.8	0.84 (0.71‐1.00)	0.84 (0.71‐1.01)
Manager	3.7	3.5	1.00 (0.87‐1.16)	1.00 (0.87‐1.15)
Service industry				
Blue‐collar	3.2	3.4	0.94 (0.77‐1.15)	0.94 (0.77‐1.15)
Service	11.5	11.2	0.98 (0.88‐1.08)	0.98 (0.88‐1.08)
Professional	1.2	1.0	1.25 (0.91‐1.71)	1.25 (0.91‐1.71)
Manager	2.0	1.7	1.12 (0.95‐1.31)	1.12 (0.95‐1.32)
White‐collar industry				
Blue‐collar	2.0	2.0	1.08 (0.87‐1.34)	1.08 (0.87‐1.34)
Service	7.2	6.8	0.93 (0.80‐1.08)	0.93 (0.80‐1.08)
Professional	5.3	4.3	1.14 (1.01‐1.29)	1.15 (1.02‐1.29)
Manager	1.2	0.9	0.99 (0.80‐1.23)	0.99 (0.80‐1.23)
Others				
Others	15.7	14.0	0.94 (0.87‐1.01)	0.94 (0.87‐1.01)
*All sites*				
Before 2003	n = 523 818	n = 108 858		
Blue‐collar industry				
Blue‐collar	32.7	33.9	1.00	1.00
Service	9.9	10.0	0.98 (0.96‐1.00)	0.97 (0.95‐1.00)
Professional	2.6	2.4	0.92 (0.87‐0.96)	0.92 (0.87‐0.96)
Manager	4.7	4.9	0.94 (0.90‐0.98)	0.92 (0.88‐0.96)
Service industry				
Blue‐collar	2.6	2.6	0.98 (0.94‐1.02)	0.98 (0.94‐1.02)
Service	9.9	9.8	0.93 (0.91‐0.95)	0.93 (0.91‐0.95)
Professional	0.6	0.7	0.84 (0.78‐0.90)	0.85 (0.80‐0.92)
Manager	2.2	2.4	0.83 (0.78‐0.89)	0.82 (0.77‐0.88)
White‐collar industry				
Blue‐collar	1.9	1.7	0.88 (0.83‐0.93)	0.88 (0.83‐0.93)
Service	6.5	5.9	0.88 (0.85‐0.91)	0.88 (0.85‐0.91)
Professional	4.7	4.2	0.83 (0.80‐0.86)	0.87 (0.84‐0.90)
Manager	1.7	1.5	0.79 (0.73‐0.85)	0.80 (0.74‐0.87)
Others				
Others	20.0	19.9	0.83 (0.81‐0.85)	0.87 (0.85‐0.89)
After 2003	n = 502 429	n = 105 265		
Blue‐collar industry				
Blue‐collar	30.9	33.3	1.00	1.00
Service	12.3	12.8	0.99 (0.96‐1.01)	0.98 (0.95‐1.01)
Professional	4.0	3.9	0.91 (0.87‐0.96)	0.92 (0.87‐0.97)
Manager	3.6	3.6	1.02 (0.98‐1.06)	1.01 (0.97‐1.04)
Service industry				
Blue‐collar	3.3	3.4	0.95 (0.90‐0.99)	0.95 (0.91‐1.00)
Service	11.4	11.3	0.96 (0.94‐0.99)	0.97 (0.95‐1.00)
Professional	1.2	1.1	1.04 (0.94‐1.16)	1.06 (0.95‐1.17)
Manager	1.9	1.7	1.02 (0.96‐1.08)	1.03 (0.97‐1.09)
White‐collar industry				
Blue‐collar	1.9	1.8	0.91 (0.85‐0.98)	0.92 (0.86‐0.99)
Service	7.2	6.8	0.88 (0.85‐0.91)	0.89 (0.86‐0.93)
Professional	5.3	4.8	0.88 (0.84‐0.93)	0.91 (0.87‐0.96)
Manager	1.2	1.0	0.84 (0.79‐0.89)	0.85 (0.80‐0.90)
Others				
Others	15.7	14.5	0.94 (0.92‐0.97)	0.97 (0.95‐1.00)

CI, confidence interval; OR, odds ratio.

a Data were estimated with five imputed datasets. Percentages may not total 100 because of rounding with multiple imputation.

b Conditional logistic regression with multiple imputation, matched for age, admission date, and admitting hospital (model 1); additional adjustment for smoking and alcohol consumption (model 2).

## 4 DISCUSSION

### 4.1 All cancer sites

In Western countries, overall male cancer incidence has shown a slightly inverse socioeconomic gradient (reduced risk with higher occupational class).^3^ Focusing on the odds ratios for cancer incidence in higher‐SES groups (ie managers and professionals) across industrial clusters, we observed an inverse socioeconomic gradient in Japan, explained by reduced incidence among higher occupational class groups for stomach, lung, liver, esophagus, and bladder cancer, as well as malignant lymphoma.

### 4.2 Inverse occupational gradient

Although smoking and alcohol consumption may substantially mediate the inverse socioeconomic gradient for stomach and lung cancer in Western countries,^3^, ^4^, ^21^ controlling for these behaviors did not fully explain the inverse gradients in the present study. This pattern concurs with the inverse socioeconomic gradient for female stomach and lung cancer incidence in Japan we found in a previous study (eg ORs for managers in blue‐collar industries were 0.67 for stomach cancer and 0.40 for lung cancer).^10^ Therefore, irrespective of sex differences, other factors, such as dietary habits (high salt diet) and *H. pylori* infection for stomach cancer and occupational/industrial differences in environmental exposure for lung cancer, may play a role.^10^, ^22^ Indeed, blue‐collar workers in white‐collar industries, as well as service workers in all industrial clusters, showed lower odds ratios for lung cancer risk compared with blue‐collar workers in blue‐collar industries, which also suggests the occupational and industrial differences in environmental exposure to unknown hazardous substance and/or to passive smoking in the workplace linked to lung cancer risk.

Studies in western settings have found an inverse socioeconomic gradient for esophagus cancer (as we did), while gradients for liver and pancreas cancer have been less clear.^3^, ^4^, ^21^ We observed a reduced risk with higher occupational class for esophagus and liver cancer, as well as a potentially lower risk among higher‐status occupations for pancreas cancer, even after controlling for behavioral risk factors. Dietary habits (vegetables and fruits) may be associated with a reduced risk for these cancers; however, the protective effect remains controversial in the Japanese population.^23^ As we observed a reduced liver cancer risk not only in high‐occupational class but also in white‐collar industries regardless of occupational class, socioeconomic disparities in *Hepatitis C* infection may additionally contribute to the observed socioeconomic gradients in liver cancer.^4^ A socioeconomic gradient for bladder cancer and malignant lymphoma has not been consistently observed in Western countries,^3^ while we found an inverse socioeconomic gradient. Our findings may be attributable to exposure to aromatic amines in certain high‐risk occupation (for bladder cancer)^13^, ^14^ as well as the use of pesticides (in the case of malignant lymphoma).^24^ Among women in Japan, a socioeconomic gradient was not observed for esophagus, liver, pancreas, bladder cancer, and malignant lymphoma.^10^ These differences between men and women regarding socioeconomic patterns may imply a possible sex difference in occupational roles in the same job category^19^; however, other relevant reasons remain unclear.

Evidence for socioeconomic gradients for less common cancers remains sparse.^3^

### 4.3 Null occupational gradient

The positive socioeconomic gradient for colon cancer has been reported in Western countries.^3^, ^4^ The incidence of colorectal cancer has dramatically increased in Japan since the 1970s; the age‐standardized incidence rate is now similar to that in the USA.^25^ However, we observed a null socioeconomic gradient for male colorectal cancer, as well as for female colorectal cancer in a previous study,^10^ which might be partly attributable to potential protective effects of traditional dietary habits in Japan (fish).^26^

### 4.4 Positive occupational gradient

For prostate cancer, our observed excess risk with higher occupational class has not been consistently reported worldwide,^3^ whereas an excess risk with higher occupational class, possibly related to prostate cancer screening and over‐diagnosis, has been reported in USA.^27^ In Japan, annual health checkups are conducted in the workplace,^10^ which often include an opportunity for prostate cancer screening. Therefore, those in the “other” occupational group (such as the unemployed), who are not actively engaged in paid employment, may not have had a chance for undergoing prostate cancer screening and therefore may have a lower likelihood for over‐diagnosis (Table 1); however, empirical evidence for prostate cancer screening in the Japanese population has not been reported yet.^28^

Evidence for socioeconomic gradients for kidney cancer remains sparse.^3^ An observed tendency toward a positive socioeconomic gradient for kidney cancer may be partly associated with risk of renal cell carcinoma in higher occupational class men in Japan.^11^

### 4.5 Strengths and limitations

As far as we aware, we first found the association of occupational class (as an indicator for SES) and risk of various male cancer incidence in Japan. This study is one of the largest studies for cancer incidence reported in that country. The strengths include accurate diagnosis, which was directly extracted from medical charts in contrast to less accurate diagnosis with claims data,^16^ and use of the longest‐held occupation, which is more accurate to measure SES compared with the most recent occupation.^6^, ^7^

However, some limitations should be noted. First, the selection of hospital controls might have introduced selection bias in either direction (toward or away from null). The absence of relevant population‐based data did not allow us to obtain population‐based controls (as in studies in the Nordic Occupational Cancer Study),^29^, ^30^ and one‐third of the missing information may reflect selection bias even though we performed multiple imputation. In addition, because the duration of occupation was collected at the questionnaire, recall bias might have introduced. However, occupational profiles of our controls are nationally representative,^10^, ^11^ and sensitivity analysis showed the same result. Second, other relevant socioeconomic factors (ie educational attainment and income levels)^21^ were not evaluated owing to the limitations of our data. However, a previous large‐scale study in Finland showed that male cancer incidence differed across occupational classes even within strata of educational attainment and income levels.^4^ Finally, our broad occupational category was not designed to detect occupational exposure and differed from occupational categories to detect specific occupational exposure.^29^, ^30^ In addition, we could not assess multiple primary cancer cases or other possible risk factors (overweight, diet, institutional place‐based discrimination, physical activity, and cancer screening program).^31^, ^32^, ^33^, ^34^ Therefore, future studies are warranted to integrate all these aspects of cancer causal pathways.

In conclusion, we have documented socioeconomic inequalities in risk of various male cancer incidence in Japan, which were not explained by smoking and alcohol consumption. The national cancer prevention strategy needs to explicitly incorporate strategies to address occupational class. Since national legislation to restrict indoor smoking has yet to be established in Japan, intensive promotion of preventing passive smoking in (although not limited to) the workplace should be a priority.

## CONFLICT OF INTEREST

None declared.
