# Supplementary material for: Occupational class and male cancer incidence: Nationwide, multicenter, hospital‐based case–control study in Japan
Source: Cancer Med. 2019 Jan 4;8(2):795–813. doi: 10.1002/cam4.1945 (PMC6382925; doi:10.1002/cam4.1945)
Supplement: Supplementary file 1 [file CAM4-8-795-s001.docx]

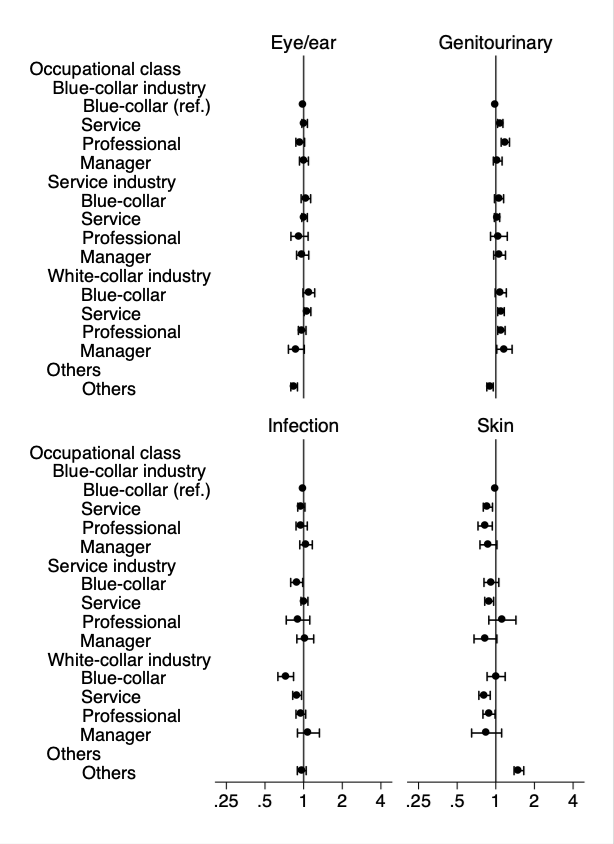
**Figure S1. Association between occupational class and each benign disease selected for controls.** Socioeconomic gradients were estimated with unconditional logistic regression adjusted for age, admission date, and admitting hospital among 89,412 subjects diagnosed with the following benign diseases: eye or ear diseases (n = 35,880), genitourinary diseases (n = 33,138), infection (n = 12,828); and skin diseases (n = 7,566). The odds ratio for each disease was estimated against the reminder of four diseases. A consistent socioeconomic gradient pattern (reduced or excess risk with higher socioeconomic status [professionals and managers]) was not observed.

**Figure S2. Longest-held occupational class cross-classified with industrial cluster**

**
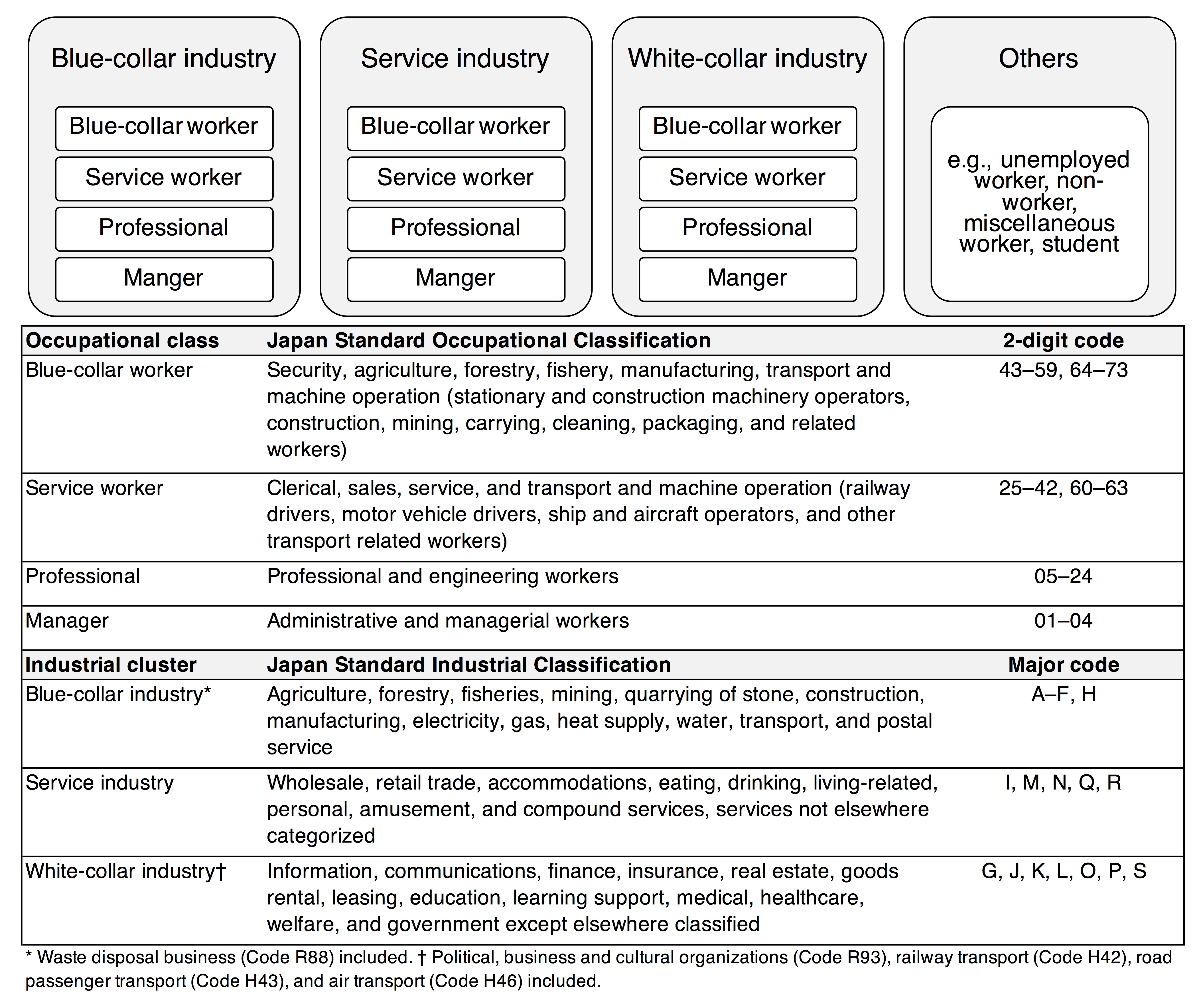
**

**Figure S3. Occupational gradients for stomach, lung, prostate, and overall cancer incidence estimated with complete data.** The odds ratio (dot) and 95% confidence interval (bar) were estimated by (a) conditional logistic regression matched for age, admission date, and admitting hospital, and (b) multilevel logistic regression with random intercepts of hospital (level 1, individual; level 2, hospital), adjusted for age and admission date. Both regression analyses were additionally adjusted for smoking and alcohol consumption. The numbers of cases and controls used for analysis were, respectively, 24,078 and 106,463 for stomach cancer, 13,075 and 57,177 for lung cancer, 18,634 and 85,665 for prostate cancer, and 125,342 and 559,198 for overall cancer. Stomach, lung, and overall cancer showed a reduced risk with higher occupational class (professionals and managers). By contrast, prostate cancer showed an excess risk with higher occupational class.


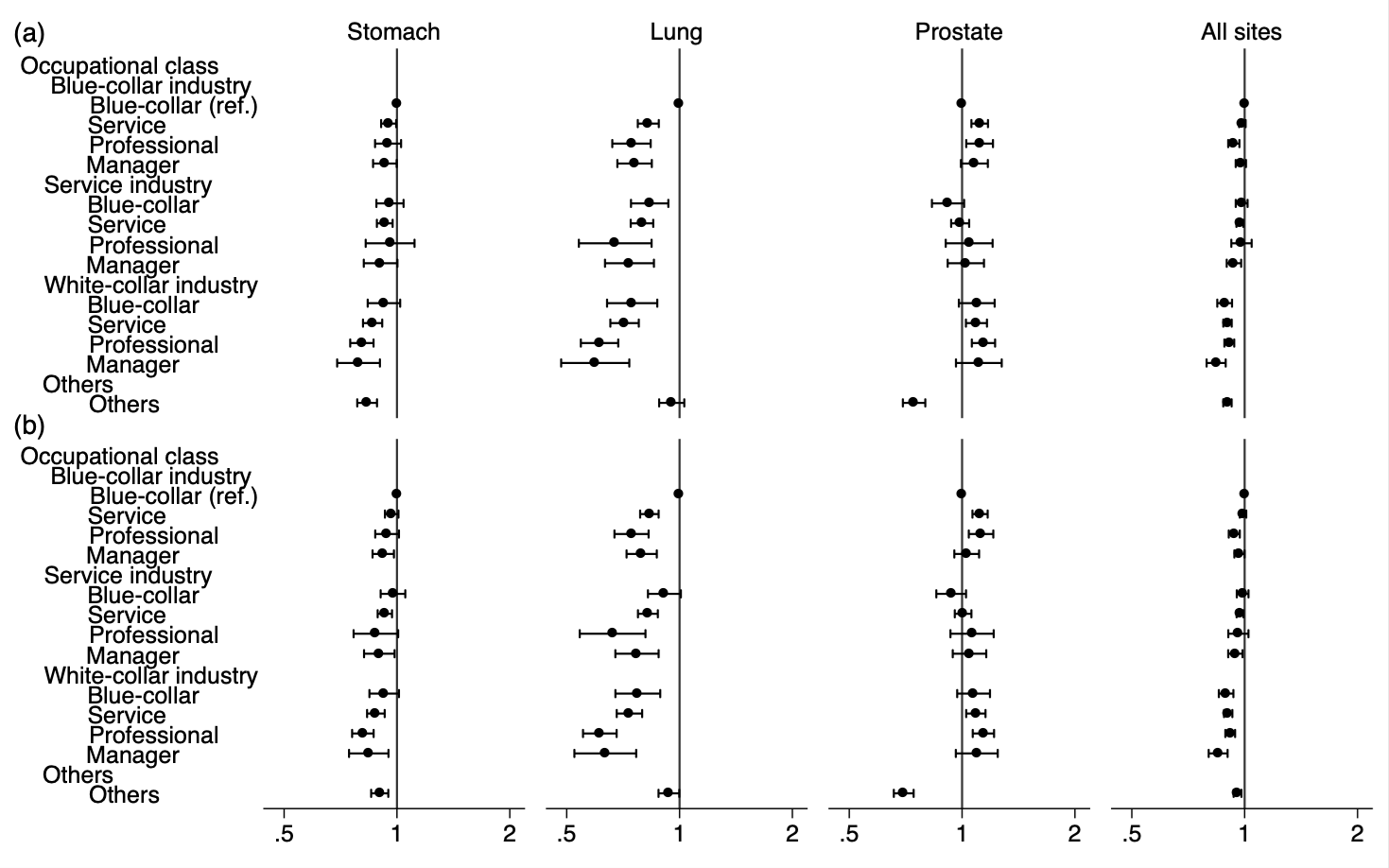


**Table S1. Prevalence of analyzed cancer cases compared to national statistics**

| Rank | Primary sites | ICD-9 | ICD-10 | Year 1984–2016 | | Year 1984–2013 | | | | Year 2005 | | | | Year 2009 | | | |
| --- | --- | --- | --- | --- | --- | --- | --- | --- | --- | --- | --- | --- | --- | --- | --- | --- | --- |
|  |  |  |  | ICOD-R | | National statistics^a^ | | ICOD-R | | National statistics^b^ | | ICOD-R | | National statistics^c^ | | ICOD-R | |
|  |  |  |  | N | % | N | % | N | % | N | % | N | % | N | % | N | % |
| 1 | Stomach | 151 | C16 | 42,510 | 19.9 | 2,166,830 | 22.7 | 39,980 | 20.2 | 80,102 | 21.1 | 1,493 | 17.4 | 84,563 | 18.7 | 1,340 | 16.7 |
| 2 | Lung | 162 | C33–34 | 21,922 | 10.2 | 1,453,806 | 15.2 | 20,087 | 10.2 | 58,264 | 15.4 | 884 | 10.3 | 71,722 | 15.9 | 866 | 10.8 |
| 3 | Colorectum | 153,154.0/154.1 | C18–20 | 27,074 | 12.6 | 1,501,393 | 15.7 | 24,560 | 12.4 | 59,470 | 15.7 | 1,179 | 13.7 | 66,859 | 14.8 | 1,170 | 14.6 |
| 4 | Prostate | 185 | C61 | 28,392 | 13.3 | 775,464 | 8.1 | 25,000 | 12.6 | 42,997 | 11.3 | 1,536 | 17.9 | 59,627 | 13.2 | 1,490 | 18.6 |
| 5 | Liver | 155 | C22 | 18,354 | 8.6 | 787,436 | 8.3 | 17,502 | 8.9 | 28,729 | 7.6 | 705 | 8.2 | 31,915 | 7.1 | 617 | 7.7 |
| 6 | Esophagus | 150 | C15 | 6,317 | 3.0 | 373,310 | 3.9 | 5,912 | 3.0 | 14,818 | 3.9 | 226 | 2.6 | 17,492 | 3.9 | 203 | 2.5 |
| 7 | Pancreas | 157 | C25 | 4,976 | 2.3 | 340,136 | 3.6 | 4,560 | 2.3 | 13,108 | 3.5 | 205 | 2.4 | 16,584 | 3.7 | 163 | 2.0 |
| 8 | Bladder | 188 | C67 | 13,590 | 6.3 | 307,363 | 3.2 | 12,540 | 6.3 | 12,619 | 3.3 | 542 | 6.3 | 14,326 | 3.2 | 509 | 6.3 |
| 9 | Kidney, pelvis & ureter | 189.0–189.2 | C64–66 | 5,552 | 2.6 | 239,765 | 2.5 | 5,024 | 2.5 | 9,758 | 2.6 | 221 | 2.6 | 12,922 | 2.9 | 248 | 3.1 |
| 10 | Malignant lymphoma | 200–202 | C81–85,96 | 6,157 | 2.9 | 240,233 | 2.5 | 5,548 | 2.8 | 8,993 | 2.4 | 223 | 2.6 | 12,074 | 2.7 | 238 | 3.0 |
| 11 | Gallbladder & bile duct | 156 | C23–24 | 3,396 | 1.6 | 243,825 | 2.6 | 3,021 | 1.5 | 9,237 | 2.4 | 186 | 2.2 | 11,419 | 2.5 | 175 | 2.2 |
| 12 | Oral cavity, pharynx | 140–149 | C00–14 | 4,031 | 1.9 | 209,912 | 2.2 | 3,755 | 1.9 | 7,417 | 2.0 | 141 | 1.6 | 10,689 | 2.4 | 131 | 1.6 |
| 13 | All leukemias | 204–208 | C91–95 | 2,586 | 1.2 | 133,161 | 1.4 | 2,391 | 1.2 | 5,200 | 1.4 | 67 | 0.8 | 6837 | 1.5 | 74 | 0.9 |
| 14 | Skin | 172–173 | C43–44 | 2,655 | 1.2 | 124,551 | 1.3 | 2,398 | 1.2 | 4,405 | 1.2 | 106 | 1.2 | 6,656 | 1.5 | 122 | 1.5 |
| 15 | Larynx | 161 | C32 | 2,526 | 1.2 | 102,360 | 1.1 | 2,367 | 1.2 | 3,903 | 1.0 | 109 | 1.3 | 4,257 | 0.9 | 85 | 1.1 |
| 16 | Thyroid | 193 | C73 | 946 | 0.4 | 57,925 | 0.6 | 882 | 0.4 | 2,126 | 0.6 | 45 | 0.5 | 3,503 | 0.8 | 30 | 0.4 |
| 17 | Multiple myeloma | 203 | C88,90 | 1,530 | 0.7 | 63,167 | 0.7 | 1,420 | 0.7 | 2,242 | 0.6 | 48 | 0.6 | 3211 | 0.7 | 53 | 0.7 |
| 18 | Brain & nerve system | 191–192 | C70–72 | 2,782 | 1.3 | 60,506 | 0.6 | 2,674 | 1.4 | NA |  | 64 | 0.7 | 2,741 | 0.6 | 54 | 0.7 |
|  | All sites | 140–208 | C00–97 | 214,123 | 100 | 9,544,573 | 100 | 197,636 | 100 | 379,436 | 100 | 8,602 | 100 | 452,268 | 100 | 8,027 | 100 |

^a^ Data extracted from the estimated cancer cases aged 20 and older by Cancer Information Service, National Cancer Center, Japan 2015 (Cancer Registry and Statistics. Cancer Information Service, National Cancer Center, Japan. 2015. https://ganjoho.jp/reg_stat/statistics/dl/index.html#incidence. Accessed on June 6, 2018). ^b^ Data extracted from Inoue M, et al. 2012 (Attributable causes of cancer in Japan in 2005--systematic assessment to estimate current burden of cancer attributable to known preventable risk factors in Japan. Ann Oncol. 2012;23:1362-1369). ^c^ Data extracted from Hori M, et al. 2015 (Cancer incidence and incidence rates in Japan in 2009: a study of 32 population-based cancer registries for the Monitoring of Cancer Incidence in Japan (MCIJ) project. Jpn J Clin Oncol. 2015;45:884-891). ICD, International Classification of Diseases; ICOD-R, Inpatient Clinico-Occupational Database of Rosai Hospital Group; NA, not available.

**Table S2. Odds ratios associated with risk for less common cancer incidence**

| Occupational class | | Control, %^a^ | Case, %^a^ | Model 1^b^ | | Model 2^c^ | |
| --- | --- | --- | --- | --- | --- | --- | --- |
|  |  |  |  | OR (95% CI) | *P* | OR (95% CI) | *P* |
| *Oral cavity, pharynx* |  | n=19,686 | n=4,031 |  |  |  |  |
| Blue-collar industry | Blue-collar | 30.1 | 31.0 | 1.00 |  | 1.00 |  |
|  | Service | 11.5 | 13.4 | 1.14 (1.02−1.28) | .03 | 1.12 (0.99−1.27) | .06 |
|  | Professional | 3.6 | 3.3 | 0.89 (0.71−1.11) | .30 | 0.90 (0.72−1.13) | .38 |
|  | Manager | 4.5 | 4.5 | 0.97 (0.80−1.16) | .73 | 0.94 (0.77−1.14) | .52 |
| Service industry | Blue-collar | 3.2 | 3.2 | 0.98 (0.80−1.21) | .87 | 1.00 (0.81−1.23) | .99 |
|  | Service | 11.2 | 12.0 | 1.06 (0.94−1.19) | .37 | 1.07 (0.95−1.21) | .29 |
|  | Professional | 0.9 | 0.8 | 0.87 (0.52−1.46) | .58 | 0.90 (0.54−1.52) | .69 |
|  | Manager | 2.2 | 2.2 | 1.00 (0.77−1.30) | .98 | 1.02 (0.79−1.32) | .89 |
| White-collar industry | Blue-collar | 2.0 | 2.1 | 1.00 (0.78−1.28) | .97 | 1.01 (0.78−1.30) | .95 |
|  | Service | 7.5 | 6.4 | 0.83 (0.70−0.98) | .03 | 0.85 (0.72−1.01) | .06 |
|  | Professional | 5.5 | 4.6 | 0.81 (0.67−0.98) | .03 | 0.88 (0.72−1.07) | .19 |
|  | Manager | 1.5 | 1.3 | 0.79 (0.55−1.15) | .22 | 0.81 (0.56−1.16) | .24 |
| Others | Others | 16.3 | 15.3 | 0.89 (0.79−0.99) | .04 | 0.96 (0.86−1.08) | .48 |
| *Gallbladder & bile duct* |  | n=15,827 | n=3,396 |  |  |  |  |
| Blue-collar industry | Blue-collar | 32.4 | 34.2 | 1.00 |  | 1.00 |  |
|  | Service | 11.3 | 10.9 | 0.93 (0.81−1.06) | .26 | 0.92 (0.81−1.06) | .24 |
|  | Professional | 3.2 | 2.5 | 0.74 (0.56−0.98) | .04 | 0.73 (0.55−0.97) | .03 |
|  | Manager | 3.8 | 3.9 | 0.97 (0.79−1.20) | .81 | 0.96 (0.78−1.18) | .71 |
| Service industry | Blue-collar | 2.8 | 2.8 | 0.97 (0.75−1.24) | .79 | 0.97 (0.75−1.25) | .83 |
|  | Service | 10.9 | 10.5 | 0.92 (0.80−1.06) | .24 | 0.92 (0.80−1.06) | .26 |
|  | Professional | 1.0 | 0.9 | 0.92 (0.57−1.48) | .73 | 0.92 (0.58−1.48) | .73 |
|  | Manager | 1.9 | 1.6 | 0.79 (0.52−1.18) | .24 | 0.79 (0.52−1.18) | .24 |
| White-collar industry | Blue-collar | 1.8 | 1.7 | 0.94 (0.70−1.26) | .68 | 0.93 (0.69−1.25) | .61 |
|  | Service | 6.1 | 5.7 | 0.90 (0.74−1.09) | .26 | 0.90 (0.74−1.08) | .26 |
|  | Professional | 4.9 | 4.7 | 0.94 (0.76−1.15) | .53 | 0.95 (0.78−1.16) | .63 |
|  | Manager | 1.3 | 1.3 | 0.95 (0.63−1.43) | .82 | 0.95 (0.63−1.44) | .81 |
| Others | Others | 18.7 | 19.4 | 0.95 (0.83−1.08) | .42 | 0.97 (0.85−1.10) | .61 |
| *Larynx* |  | n=12,410 | n=2,526 |  |  |  |  |
| Blue-collar industry | Blue-collar | 31.2 | 31.4 | 1.00 |  | 1.00 |  |
|  | Service | 10.9 | 13.4 | 1.24 (1.06−1.44) | .007 | 1.22 (1.03−1.43) | .02 |
|  | Professional | 3.4 | 2.9 | 0.85 (0.62−1.16) | .31 | 0.86 (0.63−1.17) | .32 |
|  | Manager | 4.3 | 5.3 | 1.24 (1.00−1.53) | .05 | 1.16 (0.93−1.45) | .18 |
| Service industry | Blue-collar | 3.0 | 2.2 | 0.75 (0.53−1.05) | .09 | 0.76 (0.54−1.07) | .12 |
|  | Service | 10.4 | 11.3 | 1.08 (0.88−1.33) | .44 | 1.08 (0.87−1.34) | .45 |
|  | Professional | 0.9 | 0.9 | 1.05 (0.62−1.77) | .86 | 1.07 (0.63−1.83) | .79 |
|  | Manager | 2.5 | 2.4 | 0.93 (0.69−1.26) | .65 | 0.97 (0.71−1.31) | .82 |
| White-collar industry | Blue-collar | 1.9 | 1.7 | 0.94 (0.65−1.35) | .73 | 0.91 (0.62−1.33) | .63 |
|  | Service | 7.0 | 7.3 | 1.04 (0.85−1.27) | .72 | 1.07 (0.86−1.32) | .53 |
|  | Professional | 4.9 | 3.8 | 0.78 (0.58−1.06) | .11 | 0.88 (0.65−1.19) | .41 |
|  | Manager | 1.6 | 1.3 | 0.80 (0.52−1.23) | .30 | 0.83 (0.54−1.25) | .36 |
| Others | Others | 18.2 | 16.0 | 0.84 (0.66−1.07) | .14 | 0.91 (0.72−1.16) | .42 |
| *Skin* |  | n=12,451 | n=2,655 |  |  |  |  |
| Blue-collar industry | Blue-collar | 30.8 | 31.1 | 1.00 |  | 1.00 |  |
|  | Service | 10.5 | 10.1 | 0.98 (0.79−1.21) | .82 | 0.97 (0.79−1.20) | .80 |
|  | Professional | 3.1 | 3.3 | 1.08 (0.81−1.45) | .60 | 1.08 (0.81−1.45) | .59 |
|  | Manager | 4.2 | 3.5 | 0.84 (0.61−1.14) | .25 | 0.83 (0.61−1.13) | .24 |
| Service industry | Blue-collar | 2.8 | 2.8 | 1.03 (0.73−1.46) | .86 | 1.03 (0.73−1.45) | .86 |
|  | Service | 10.5 | 10.7 | 1.03 (0.87−1.22) | .75 | 1.03 (0.87−1.22) | .74 |
|  | Professional | 0.9 | 0.9 | 1.05 (0.62−1.76) | .86 | 1.05 (0.63−1.76) | .85 |
|  | Manager | 2.0 | 1.9 | 0.94 (0.64−1.38) | .75 | 0.94 (0.64−1.38) | .75 |
| White-collar industry | Blue-collar | 1.7 | 1.4 | 0.78 (0.49−1.26) | .30 | 0.79 (0.49−1.26) | .31 |
|  | Service | 6.6 | 6.8 | 1.04 (0.79−1.38) | .77 | 1.04 (0.78−1.38) | .77 |
|  | Professional | 5.2 | 4.9 | 0.96 (0.77−1.20) | .74 | 0.98 (0.78−1.22) | .84 |
|  | Manager | 1.4 | 0.9 | 0.62 (0.39−1.00) | .05 | 0.62 (0.39−1.00) | .05 |
| Others | Others | 20.3 | 21.6 | 1.03 (0.90−1.17) | .65 | 1.04 (0.92−1.19) | .52 |
| *Brain & nerve system* |  | n=13,408 | n=2,782 |  |  |  |  |
| Blue-collar industry | Blue-collar | 32.0 | 32.3 | 1.00 |  | 1.00 |  |
|  | Service | 11.8 | 11.0 | 0.92 (0.80−1.07) | .30 | 0.92 (0.80−1.07) | .31 |
|  | Professional | 4.0 | 3.3 | 0.83 (0.59−1.16) | .26 | 0.83 (0.59−1.16) | .26 |
|  | Manager | 4.0 | 4.2 | 1.05 (0.79−1.41) | .71 | 1.06 (0.79−1.41) | .70 |
| Service industry | Blue-collar | 3.4 | 3.2 | 0.93 (0.69−1.25) | .62 | 0.93 (0.69−1.25) | .62 |
|  | Service | 12.0 | 12.4 | 1.02 (0.87−1.21) | .77 | 1.02 (0.87−1.21) | .78 |
|  | Professional | 0.8 | 0.8 | 1.04 (0.62−1.75) | .88 | 1.04 (0.62−1.76) | .88 |
|  | Manager | 1.9 | 2.1 | 1.11 (0.75−1.63) | .59 | 1.11 (0.75−1.63) | .59 |
| White-collar industry | Blue-collar | 2.0 | 2.4 | 1.17 (0.81−1.67) | .39 | 1.17 (0.82−1.67) | .38 |
|  | Service | 7.6 | 6.5 | 0.85 (0.66−1.10) | .20 | 0.85 (0.66−1.10) | .20 |
|  | Professional | 5.4 | 5.7 | 1.06 (0.86−1.30) | .61 | 1.05 (0.85−1.30) | .62 |
|  | Manager | 1.6 | 1.2 | 0.78 (0.53−1.16) | .23 | 0.78 (0.53−1.17) | .23 |
| Others | Others | 13.6 | 14.9 | 1.08 (0.92−1.26) | .35 | 1.07 (0.92−1.26) | .38 |
| *Thyroid* |  | n=4,434 | n=946 |  |  |  |  |
| Blue-collar industry | Blue-collar | 30.5 | 30.3 | 1.00 |  | 1.00 |  |
|  | Service | 11.9 | 11.8 | 1.01 (0.77−1.33) | .93 | 1.01 (0.77−1.34) | .92 |
|  | Professional | 4.2 | 5.8 | 1.37 (0.97−1.94) | .07 | 1.37 (0.97−1.93) | .08 |
|  | Manager | 4.0 | 5.0 | 1.26 (0.87−1.83) | .22 | 1.26 (0.87−1.82) | .22 |
| Service industry | Blue-collar | 3.6 | 3.5 | 0.98 (0.64−1.50) | .93 | 0.96 (0.63−1.47) | .86 |
|  | Service | 11.6 | 9.5 | 0.83 (0.63−1.11) | .21 | 0.83 (0.62−1.10) | .19 |
|  | Professional | 1.0 | 0.6 | 0.55 (0.20−1.49) | .24 | 0.54 (0.20−1.47) | .23 |
|  | Manager | 2.3 | 1.9 | 0.90 (0.52−1.58) | .72 | 0.90 (0.51−1.57) | .70 |
| White-collar industry | Blue-collar | 2.2 | 2.7 | 1.29 (0.75−2.22) | .34 | 1.30 (0.76−2.24) | .34 |
|  | Service | 7.7 | 7.5 | 0.99 (0.70−1.41) | .96 | 0.99 (0.70−1.41) | .97 |
|  | Professional | 5.9 | 7.5 | 1.29 (0.89−1.86) | .17 | 1.26 (0.87−1.84) | .22 |
|  | Manager | 1.4 | 0.8 | 0.54 (0.23−1.29) | .17 | 0.53 (0.22−1.27) | .15 |
| Others | Others | 13.8 | 13.1 | 0.88 (0.68−1.15) | .35 | 0.87 (0.67−1.13) | .28 |
| *Multiple myeloma* |  | n=7,285 | n=1,530 |  |  |  |  |
| Blue-collar industry | Blue-collar | 31.4 | 36.1 | 1.00 |  | 1.00 |  |
|  | Service | 10.7 | 10.1 | 0.82 (0.64−1.05) | .12 | 0.82 (0.64−1.05) | .11 |
|  | Professional | 3.4 | 3.0 | 0.77 (0.52−1.15) | .19 | 0.77 (0.52−1.15) | .20 |
|  | Manager | 4.1 | 3.9 | 0.82 (0.58−1.15) | .25 | 0.82 (0.58−1.15) | .24 |
| Service industry | Blue-collar | 2.8 | 2.5 | 0.78 (0.53−1.13) | .18 | 0.77 (0.53−1.12) | .17 |
|  | Service | 10.5 | 10.8 | 0.88 (0.70−1.11) | .27 | 0.88 (0.69−1.11) | .26 |
|  | Professional | 0.8 | 0.7 | 0.82 (0.32−2.14) | .67 | 0.83 (0.32−2.15) | .68 |
|  | Manager | 2.1 | 1.8 | 0.73 (0.46−1.15) | .17 | 0.73 (0.46−1.15) | .17 |
| White-collar industry | Blue-collar | 1.8 | 1.4 | 0.66 (0.36−1.22) | .17 | 0.66 (0.36−1.23) | .18 |
|  | Service | 6.9 | 5.5 | 0.70 (0.51−0.96) | .03 | 0.70 (0.51−0.96) | .03 |
|  | Professional | 4.8 | 3.7 | 0.67 (0.47−0.95) | .02 | 0.68 (0.48−0.96) | .03 |
|  | Manager | 1.5 | 1.3 | 0.75 (0.44−1.28) | .29 | 0.75 (0.44−1.29) | .30 |
| Others | Others | 19.3 | 19.4 | 0.87 (0.73−1.03) | .10 | 0.87 (0.73−1.03) | .11 |
| *All leukemias* |  | n=14,492 | n=3,029 |  |  |  |  |
| Blue-collar industry | Blue-collar | 31.4 | 32.8 | 1.00 |  | 1.00 |  |
|  | Service | 11.2 | 11.1 | 0.96 (0.78−1.18) | .68 | 0.95 (0.77−1.18) | .63 |
|  | Professional | 3.7 | 3.1 | 0.81 (0.62−1.07) | .14 | 0.82 (0.62−1.07) | .14 |
|  | Manager | 3.8 | 3.7 | 0.94 (0.71−1.24) | .63 | 0.93 (0.70−1.23) | .58 |
| Service industry | Blue-collar | 2.9 | 3.3 | 1.08 (0.85−1.38) | .52 | 1.09 (0.85−1.39) | .50 |
|  | Service | 11.0 | 11.1 | 0.97 (0.82−1.14) | .70 | 0.98 (0.83−1.15) | .76 |
|  | Professional | 0.7 | 0.9 | 1.24 (0.72−2.12) | .42 | 1.26 (0.73−2.17) | .40 |
|  | Manager | 1.9 | 2.2 | 1.10 (0.82−1.49) | .52 | 1.11 (0.82−1.49) | .51 |
| White-collar industry | Blue-collar | 2.0 | 1.8 | 0.84 (0.57−1.22) | .35 | 0.84 (0.58−1.23) | .36 |
|  | Service | 7.2 | 6.3 | 0.84 (0.65−1.07) | .15 | 0.85 (0.66−1.09) | .18 |
|  | Professional | 5.0 | 4.6 | 0.89 (0.70−1.13) | .34 | 0.91 (0.72−1.16) | .45 |
|  | Manager | 1.5 | 1.1 | 0.72 (0.40−1.32) | .27 | 0.73 (0.40−1.35) | .29 |
| Others | Others | 17.7 | 18.0 | 0.96 (0.81−1.14) | .64 | 0.98 (0.83−1.16) | .85 |

^a^ Data were estimated with five imputed datasets. Percentages may not total 100 because of rounding with multiple imputation. ^b^ Conditional logistic regression matched for age, admission date, and admitting hospital, with multiple imputation. ^c^ Additional adjustment for smoking (log-transformed pack-year) and alcohol consumption (log-transformed daily gram of ethanol intake). Gallbladder cancer, leukemia, and multiple myeloma appeared to show a possible reduced risk with higher occupational class. OR, odds ratio; CI, confidence interval.

**Table S3. Odds ratios associated with risk for lung and prostate cancer estimated with alternative control groups**

| Occupational class | | Control, %^a^ | Case, %^a^ | Model 1^b^ | | Model 2^c^ | |
| --- | --- | --- | --- | --- | --- | --- | --- |
|  |  |  |  | OR (95% CI) | *P* | OR (95% CI) | *P* |
| *Lung* |  | n=110,321 | n=22,086 |  |  |  |  |
| Blue-collar industry | Blue-collar | 34.2 | 37.4 | 1.00 |  | 1.00 |  |
|  | Service | 10.3 | 10.6 | 0.94 (0.88−1.00) | .04 | 0.93 (0.88−0.99) | .02 |
|  | Professional | 2.8 | 2.7 | 0.85 (0.77−0.94) | .002 | 0.85 (0.77−0.95) | .004 |
|  | Manager | 3.9 | 4.0 | 0.94 (0.86−1.02) | .14 | 0.92 (0.85−1.00) | .06 |
| Service industry | Blue-collar | 2.9 | 2.9 | 0.93 (0.85−1.02) | .13 | 0.94 (0.86−1.04) | .23 |
|  | Service | 9.6 | 9.3 | 0.88 (0.83−0.94) | <.001 | 0.89 (0.84−0.95) | <.001 |
|  | Professional | 0.9 | 0.7 | 0.66 (0.55−0.80) | <.001 | 0.68 (0.56−0.82) | <.001 |
|  | Manager | 2.0 | 1.8 | 0.83 (0.72−0.95) | .01 | 0.82 (0.72−0.94) | .004 |
| White-collar industry | Blue-collar | 1.8 | 1.5 | 0.79 (0.67−0.92) | .004 | 0.81 (0.69−0.95) | .01 |
|  | Service | 5.7 | 5.4 | 0.86 (0.79−0.94) | .001 | 0.89 (0.81−0.97) | .007 |
|  | Professional | 4.4 | 3.3 | 0.67 (0.61−0.75) | <.001 | 0.73 (0.65−0.81) | <.001 |
|  | Manager | 1.3 | 1.0 | 0.69 (0.58−0.82) | <.001 | 0.71 (0.60−0.84) | <.001 |
| Others | Others | 20.2 | 19.4 | 0.87 (0.83−0.92) | <.001 | 0.94 (0.89−1.00) | .04 |
| *Prostate* |  | n=143,090 | n=28,648 |  |  |  |  |
| Blue-collar industry | Blue-collar | 32.9 | 31.6 | 1.00 |  | 1.00 |  |
|  | Service | 11.1 | 12.0 | 1.13 (1.07−1.18) | <.001 | 1.13 (1.08−1.19) | <.001 |
|  | Professional | 3.1 | 3.6 | 1.21 (1.09−1.33) | <.001 | 1.21 (1.09−1.33) | <.001 |
|  | Manager | 3.9 | 4.0 | 1.07 (0.99−1.16) | .10 | 1.08 (0.99−1.17) | .08 |
| Service industry | Blue-collar | 3.0 | 2.7 | 0.94 (0.86−1.03) | .19 | 0.94 (0.86−1.02) | .15 |
|  | Service | 10.0 | 9.9 | 1.03 (0.98−1.09) | .23 | 1.03 (0.98−1.09) | .26 |
|  | Professional | 1.1 | 1.1 | 1.13 (0.99−1.28) | .06 | 1.12 (0.99−1.27) | .08 |
|  | Manager | 2.0 | 2.0 | 1.05 (0.94−1.17) | .36 | 1.05 (0.95−1.17) | .32 |
| White-collar industry | Blue-collar | 1.9 | 2.0 | 1.12 (1.00−1.25) | .04 | 1.12 (1.00−1.24) | .05 |
|  | Service | 6.0 | 6.7 | 1.17 (1.08−1.25) | <.001 | 1.16 (1.08−1.25) | <.001 |
|  | Professional | 4.5 | 5.4 | 1.24 (1.15−1.34) | <.001 | 1.22 (1.14−1.32) | <.001 |
|  | Manager | 1.2 | 1.3 | 1.13 (1.00−1.27) | .06 | 1.12 (0.99−1.27) | .07 |
| Others | Others | 19.3 | 17.6 | 0.93 (0.89−0.98) | .003 | 0.92 (0.88−0.96) | <.001 |

^a^ Data were estimated with five imputed datasets. Percentages may not total 100 because of rounding with multiple imputation. The control group comprised patients diagnosed with benign neoplasm (4.6%), circulatory disease (23.0%), digestive disease (12.2%), endocrine disease (3.5%), eye and ear disease (7.4%), genitourinary system disease (7.7%), infectious disease (2.2%), injury (12.6%), mental disease (0.6%), musculoskeletal disease (8.3%), nerve system disease (3.1%), respiratory disease (7.6%), skin diseases (1.1%), symptoms (2.1%), or other diseases such as congenital malformations (4.3%). ^b^ Conditional logistic regression matched for age, admission date, and admitting hospital, with multiple imputation. A reduced risk with higher occupational class (professionals and managers) was observed for lung cancer. By contrast, an excess risk with higher occupational class was observed for prostate cancer. ^c^ Additional adjustment for smoking (log-transformed pack-year) and alcohol consumption (log-transformed daily gram of ethanol intake). Even after controlling for smoking and alcohol consumption, the odds in higher occupational class were not attenuated and remained significantly associated with lung and prostate cancer. OR, odds ratio; CI, confidence interval.
